# Comparison between Legiolert and real time PCR in the detection of *Legionella pneumophila* from environmental water samples

**DOI:** 10.1371/journal.pone.0336207

**Published:** 2025-12-02

**Authors:** Ignazio Arrigo, Nicola Serra, Laiba Mariyam, Mandana Mirzajani Sarvandani, Maria Rita Tricoli, Roberta Palermo, Orazia Diquattro, Giuseppe Gallina, Paola Di Carlo, Alberto Firenze, Mario Palermo, Anna Giammanco, Teresa Maria Assunta Fasciana

**Affiliations:** 1 Regional Reference Laboratory for Clinical Environmental Surveillance and Control of Legionellosis, Palermo, Italy; 2 Department of Neuroscience, Reproductive Sciences and Dentistry–Audiology Section, University of Naples Federico II, Naples, Italy; 3 Department of Precision Medicine in Medical, Surgical and Critical Care (Me.Pre.C.C.), University of Palermo, Palermo, Italy; 4 Department of Precision Medicine in the Medical, Surgical and Critical Care Areas, PhD Program Precision Medicine, University of Palermo, Palermo, Italy; 5 Department of Health Promotion, Mother and Child Care, Internal Medicine and Medical Specialities, University of Palermo, Palermo, Italy; 6 Department of Prevention, Living Environment Hygiene Service, ASP Palermo, Palermo, Italy; 7 U.O.C. Microbiology and Virology, A.O. Ospedali Riuniti Villa Sofia-Cervello, Palermo, Italy; 8 Department of Precision Medicine in the Medical, Surgical and Critical Care Areas, University of Palermo, Palermo, Italy; 9 Sicilian Health Department, Public Health and Environmental Risks Service, Palermo, Italy; Maria Curie-Sklodowska University, POLAND

## Abstract

Legionellosis is a resvpiratory disease of public health concern. Identification and quantification from environmental sources are crucial for identifying outbreak origins and providing information for risk assessment and disease prevention. *Legionella pneumophila* is typically detected and quantified using the culture method, which is considered the gold standard, but it has some critical limitations. The *Legioler/Quanti-Tray* test can be used as an alternative method to simplify the testing process and reduce the time required to obtain the result. In this study, we compare the new liquid culture method *Legiolert™* and real-time PCR with traditional plate culture, assessing the performance of PCR and culture methods for detecting *L. pneumophila* in potable water samples. We analyzed 75 environmental water samples in parallel using the Standard method (ISO 11731:1998), *Legiolert*, and real-time PCR for the detection of *L. pneumophila*. The McNemar test was used to assess the difference in accuracy between the *Legiolert* and real-time PCR methods, showing that the culture test was more accurate than the molecular biology method. The study confirmed that the *Legiolert* test is specific, easy to use, and may serve as an alternative to standardized procedures for the quantification of *L. pneumophila* in water. However, due to its high sensitivity and rapid result acquisition, we believe it could be used as a screening tool to quickly ascertain the absence of the microorganism.

## 1. Introduction

*Legionella* is a genus consisting of fastidious waterborne pathogens responsible for a clinical picture in the respiratory tract, symptoms range from influenza-like disease (Pontiac fever) to severe pneumonia (Legionnaires’ disease, LD), and infection is acquired primarily through the inhalation or aspiration of contaminated water aerosols or droplets by the Gram-negative bacteria *Legionella* spp. [1]. Its pathogenicity is related to the host’s ability to exhibit pathogenic factors that influence the intracellular growth of *Legionella pneumophila* (*Lp*) in macrophages as well as in protozoa, which is also mediated by biofilm production [2]. Legionellosis cannot be spread person to-person and is only acquired from environmental sources [3]. Nowadays, more than 65 species belong to the *Legionella* genus and *Lp* is organized in 15 serogroups, in particular, serogroup 1 is responsible for approximately 80% of all *Legionella*-related infections [4]. The number of serogroups and species is constantly increasing. In many countries, the total number of cases is probably higher than the notified data due to underdiagnosis and surveillance issues [5]. In the latest 2023 annual epidemiological report for legionnaires’ disease surveillance, the incidence of legionellosis in Italy was 66.3 cases per million inhabitants, with an increment compared to the previous year (52.8/1,000,000), higher than even pre-pandemic values [6]. Monitoring *Lp* contamination of potable water systems is of essential importance for risk assessment. The density of *Lp* cells in water is theoretically associated with the risk of legionellosis, cell densities of about 10^4^ to 10^5^ CFU per liter of water have been shown to represent a potential health risk for humans [7]. Water systems of large buildings, such as hospitals, hotels, etc are often contaminated by *Lp* [8]. Therefore, a thorough understanding of *Lp* detection methods and their performance is needed to develop preventive measures. It is widely known that legionnaires disease related to exposure to man-made water contaminated systems is caused by *Legionella* proliferation in the water environment and the exposure more than 1000 CFU/L lead to a higher risk of contagion. On the bases of this consideration have been proposed different methods for the detection of *Lp,* alternatively the standard culture method according to ISO 11731:2017, performed using specific media (buffered charcoal yeast extract, BCYE) usually supplemented with antimicrobial selective substances such as glycine, vancomycin, polymyxin and cycloheximide (GVPC). This practice considered the gold standard for the detection and enumeration of *Lp* in water samples [9], allowing the isolation of all species and serogroups of *Legionella,* to carry out comparative study with clinical isolates of *Legionella* spp, presumably associated with infection, to identify the source of infection, possible if the number of environmental contaminants is high. Unfortunately, it is time consuming as the culture requires long incubation periods of up to ten days, constituting a problem in time-sensitive cases such as outbreak cases. Furthermore, this method is expensive and requires significant experience in recognizing *Lp* colonies, and the enumeration of a *Lp* concentration may be underestimated for the inability to detect *Lp* within amoebae and viable but not-culturable bacteria (VBNC), yet potentially pathogenic [10,11]. For environmental water samples, there is a promising alternative method Legiolert test, a liquid culture method based on bacterial enzyme detection technology that allows all serogroups of *Lp* to be detected in water samples. The Legiolert method based on the most probable number (MPN) enumeration to quantify *Lp* in 7 days was developed by IDEXX Laboratories, Westbrook, ME, USA. MPN is a statistical method used to estimate the viable number of bacteria in a sample and is equivalent to the CFU that is traditionally reported by plating method. The presence of *Lp* is visualized through the utilization of a substrate present in the Legiolert reagent. In previous studies, Legiolert has shown equal performance to traditional plate culture method [12]. One limitation of the Legiolert method is that it is designed to detect only *Lp*, whereas other species remain undetectable. All of the above-mentioned difficulties lead to the development of new methods for the detection and quantification of *Lp* in water samples. The research of rapid and sensitive methods for the detection and enumeration of *Lp* cells is an issue for increasing importance of water monitoring [13]. Since 2015, molecular methods such as real-time PCR were already introduced, aiming to obtain the rapid identification [14]. Molecular technique is based on the detection of *Lp* DNA, and it has been demonstrated to be highly specific and sensitive, it offers an efficient application to detect human pathogens using nucleic acid isolated from environmental samples and can detect intracellular and viable but non-cultivable bacteria. The main advantage of this technique is the ability to detect *Lp* contamination at very low levels. RT-PCR is a widely accepted alternative to detect bacterial genomes in environmental samples, in fact constitutes a rapid tool for the survey of the bacterium, risk assessment and prevention of the spread of the disease [15]. In this study we report the results of a comparison of three methods, assessing the performance of real-time PCR and culture methods for detection of *L. pneumophila* from potable water samples. In particular, it assesses the advantages/disadvantages of the new culture method Legiolert over the traditional one and the usefulness of molecular biology.

## 2. Materials and methods

### 2.1 Samples collection

Over a period of 4 months, according to the 2015 Italian guidelines for the prevention and control of legionellosis, a total of 75 water samples were collected. At each sampling point, they were collected in two sterile bottles (1100 mL each) containing sodium thiosulphate to quench residual disinfectant [16]. The hospitals and hotels investigated were in the western part of the Sicilian Island and had different sizes and ages. The principal sites of sampling were storage tanks (cold water), boilers (hot water), sinks and showers. Samples were maintained in isotherm (ambient) conditions during transport to the laboratory and processed within 24 hours.

### 2.2 Quantification of *Legionella* by culture

The ISO 11731:2017 procedure is based on the growth of presumptive *Legionella* colonies using selective, glycine, vancomycin, polymyxin, cycloheximide, agar (GVPC). The main advantage of this technique is the ability to detect contamination of all *Legionella* spp. Briefly, one-litre sample of water was concentrated by filtration using a vacuum pump, through a 0.2-μm pore size membrane (Millipore, USA) and the filter was placed in 10 mL of the original sample and re-suspended by vortex. After sample concentration, to further reduce interfering microbial growth, heat treatment (30 min at 50°C ± 1°C) in a water bath was performed in parallel with a direct inoculation of the concentrated treated sample (100 μL) and non-treated sample into GVPC agar plates. The inoculated plates were then incubated for 10 days at 37°C in a moist chamber with 5% CO_2_. Samples were observed every 2 days until the end of incubation period. Five colonies of each presumptive *Legionella* colony were simultaneously sub-cultured to buffered charcoal yeast extract (BCYE) agar plates with and without cysteine (Oxoid, UK). Isolates that grew on BCYE agar and didn’t grow on BCYE agar without cysteine were considered *Legionella*. Latex agglutination (Oxoid, UK) was performed to confirm species and serogroups. This test allows the discrimination of *Lp* serogroup 1 from serogroups 2–14 and *Legionella* spp. most isolated. The results were expressed as CFU/L, and the detection limit of the procedure was 100 CFU/L.

### 2.3 Legiolert microbiological assay

New quantitative methods have been introduced in recent years to facilitate routine testing. Legiolert is a novel liquid culture method based on the most probable number (MPN) enumeration of *L. pneumophila* developed by IDEXX Laboratories, Inc. Legiolert is a method that allows all serogroups of *L. pneumophila* to be detected in water samples. 100 mL aliquots from the water samples were pre-assessed for total water hardness level using a colorimetric indicator dip-strip (Aquadur test; Macherey-Nagel, Germany), for low water hardness (0–2 fields on the test strip) and for high water hardness (3–4 fields on the test strip), 0.33 ml and 1.0 ml of reagent from the auxiliary kit respectively was added. After water hardness analysis, samples were then transferred to a Quanti-Tray/Legiolert pouch and heat-sealed in a Quanti-Tray sealer-plus machine to contain the sample in every well of the pouch. Quanti-Tray/Legiolert was incubated with the paper side down and the wells facing up in a humidified environment at 37°C for seven days. Results were determined by visual inspection, reading positive wells (presence of brown pigmentation/turbidity) and cross-referencing values to a most-probable number table. MPN results were determined using the supplied MPN table from IDEXX. Enumeration by the MPN technique Legiolert has a detection limit of 1 MPN in 100 mL of sample. A protocol to confirm was supplied by IDEXX. Briefly, wells selected for testing were wiped with an alcohol wipe and allowed to dry. A sterile pipette tip was then used to withdraw a 5 μL, which was transferred from each well to both a BCYE agar plate and BCYE without cysteine (BCYE-). Each aliquot was streaked for isolation and plates were incubated for 48 h at 37°C in a moist chamber with 5% CO_2_. Following incubation isolates were regarded as *L. pneumophila* if they grew on BCYE but failed to grow on BCYE-.

### 2.4 Qualitative real-time PCR

Initially, 1 L samples of water were concentrated by filtration on a 0.45 µm pore diameter polycarbonate membrane (Sartorius AG, Gottingen, Germany). DNA was extracted using a commercially available kit (Diatheva, Italy). According to the manufacturer’s instructions, the membrane was transferred into a tube with a lysis buffer and treated at 95°C for 15 minutes. The samples were left at room temperature for 20 min, the supernatant was collected, and DNA was purified by adsorption on a silica column. Finally, DNA was eluted using 30 µl of elution buffer (supplied in the kit) and stored at −20 C until real-time PCR analysis. Qualitative PCR was performed with the thermocyclers QuantStudio 3 (Applied Biosystems) and a commercially available kit (DI-Check *Legionella pneumophila* kit; Diatheva). For each sample, 5 µl of DNA was mixed with 20 µl of amplification mixture. Dye-labeled probes target unique DNA sequences specific to *Lp* and synthetic internal amplification control DNA (IAC). Each PCR run consisted of a variable number of samples, a negative PCR control well (sterile ultrapure water) and a positive PCR control well (genomic DNA of *L. pneumophila* ATCC 33152). Target DNA, if present, is amplified by PCR and detected in real-time using fluorescent hydrolysis probe chemistry. PCR was performed under the following conditions: 3 min at 95°C, 45 cycles of 15 s at 95°C and 90 s at 60°C. The presence of PCR inhibitors in extracted DNA was considered if there was no amplification of the internal inhibition control. Real-time PCR amplification curves were determined using QuantStudio Analysis Software v1.4.3.

### 2.5 Statistical analysis

Data were presented as numbers and percentages for categorical variables, and continuous data were expressed as the mean and standard deviation (SD) unless otherwise specified. Legiolert and real-time PCR methods in the detection of *L. pneumophila* were compared, considering as gold standard the culture method. For this scope, we used sensitivity, specificity, and accuracy indexes. Additionally, the Receiver Operating Characteristic (ROC) curve was used. The ROC curve plots the true positive rate in function of the false positive rate at different cut-off points, and the areas under the ROC curve (AUC) were evaluated and compared. Particularly, to compare two AUCs, the z-test was used. McNemar test was used to evaluate the difference between the accuracies of Legiolert and real-time PCR methods. Finally, all tests with p-value (p) < 0.05 were considered significant. The statistical analysis was performed using the Matrix Laboratory (MATLAB) analytical toolbox version 2008 (MathWorks, Natick, MA, USA). for Windows at 32 bits.

## 3. Results

**Fig 1:**
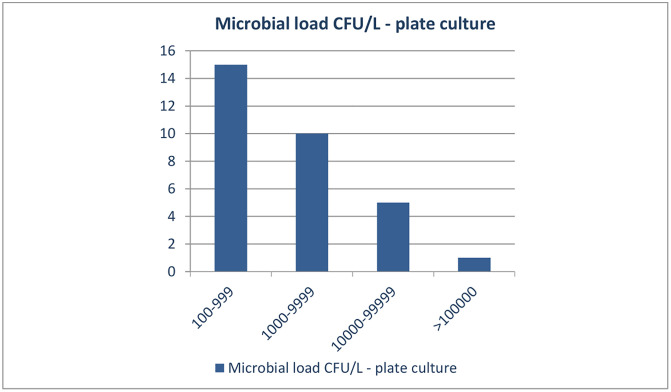
Microbial load of *Lp* detected in plate culture positive samples.

Samples were analysed over the period of 4 months, from April to July 2023. Of the 75 tested samples in parallel, 56 were positive by at least one of the methods. *Lp* was found in 42.7% (32/75), 46.6% (35/75) and 74.6% (56/75) of the samples analyzed by Standard culture method, Legiolert and real-time PCR, respectively. *Lp* was the only species isolated from the water systems of the facilities investigated. For the standard culture method, *Lp* was detected in 42.6% samples with measured concentrations ranging from 1x10^2^ CFU/L to 2,5x10^5^ CFU/L. Of this number, ten samples (31.2%) contained more than 1000 CFU/L, and 15.6% had > 10000 CFU/L (Fig 1).

**Fig 2:**
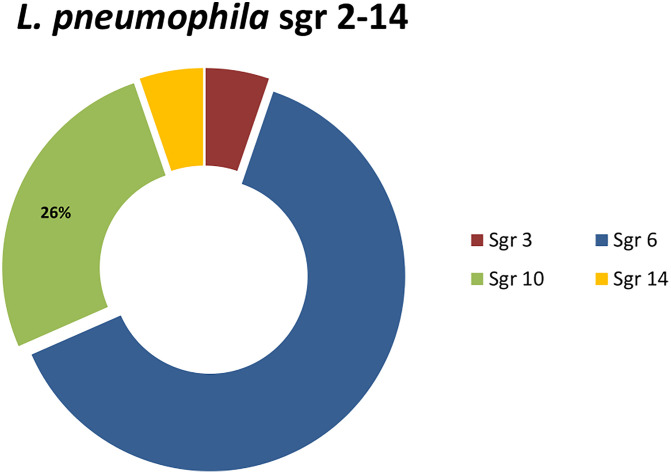
Serogroups of *Lp* detected by colonies identify on agar plate.

Consequently, no *Legionella* contamination was observed in 57.3% (43) samples with a detection limit of 100 CFU/L. Mixed *Lp* cultures (serogroup 1 and serogroup 2–14) were obtained in 7 of 31 samples. Regarding the colonies identified on the agar plates *Lp* sgr 2–14 (n°19) by agglutination test with single sera, the most represented sgr 6 (twelve samples) followed by sgr 10 (five) (Fig 2).

Finally, *Lp* sgr 3 and *Lp* sgr 14 were only detected in one case. Legiolert test results derived from 100 mL water samples (LOD = 1.0 × 10 MPN/100mL) showed that 46.6% (35) samples were positive to *Lp*, with a highly variable range from 2.2 to >2279 MPN. Thus, from the 75 samples analyzed 37.3% (28) were positive for both methods (Legiolert and standard culture).

In Table 1, we reported the results obtained with Legiolert and real-time PCR than Standard culture method.

**Table 1:** Results for the detection of *L. pneumophila* by Legiolert and real-time PCR, and plate culture method.

	Legiolert		real-time PCR	
Plate Culture	positive	negative	positive	negative
positive	28	4	32	0
negative	7	36	24	19

Specifically, we found positive samples for the Standard culture method in 42.7% (32), for Legiolert in 46.6% (35) and for real-time PCR method in 74.6% (56) samples. The present PCR results were higher than those obtained by culture. This systematic bias could be explained by the fact that culture only quantifies viable and cultivable *Lp,* underestimating the number of *Lp* cells because not detect VBNC and the cells present in protozoa.

In Table 2 we evaluate the performance of Legiolert and real-time PCR methods to detect positive culture to *Lp*, using statistical indexes such as sensitivity, specificity, and accuracy, and considering as gold standard the Standard culture method.

**Table 2:** Legiolert and real-time PCR methods performance about to detect positive culture to *Lp*.

Method	Sensitivity (%) (CI at 95%)	Specificity (%) (CI at 95%)	Accuracy (%) (CI at 95%)
Legiolert	87.5% (77.4% − 94.0%)	83.7% (73.0%, 91.2%)	85.3% (74.9%, 92.4%)
real-time PCR	100% (93.9%, 100%)	44.2% (32.9%, 56.1%)	68.0% (56.1%, 78.1%)

Table 2 shows for Legiolert high percentages of sensitivity (87.5%), specificity (83.7%), and accuracy (85.3%), while for real-time PCR we found a high sensitivity (100%), and moderate specificity (44.2%), and accuracy (68.0%). Particularly, comparing the accuracy of Legiolert and real-time PCR, we found a significant difference (85.3% vs 68%, p = 0.0106). This result shows more accuracy of Legiolert than real-time PCR method.

**Fig 3:**
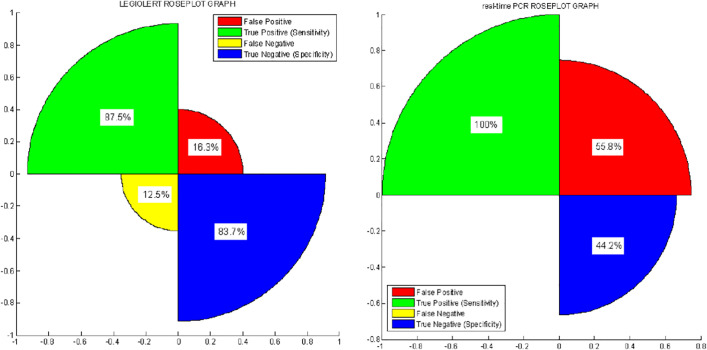
Rose-plot graph for Legiolert and real-time PCR method.

Fig 3 shows the rose-plot graph for Legiolert and real-time PCR. The rose-plot graph describes the percentages of true negative (specificity), true positive (sensitivity), false negative, and false positive. The rose-plot uses the area of segments of the circle to convey amounts, where the angle is constant, i.e., divide 360 by the number of parameters considered and it is the square root of the radius that is proportional to percentages.

Additionally, we compared the Legiolert and real-time PCR method considering the respective area under the ROC curves (AUC).

**Fig 4:**
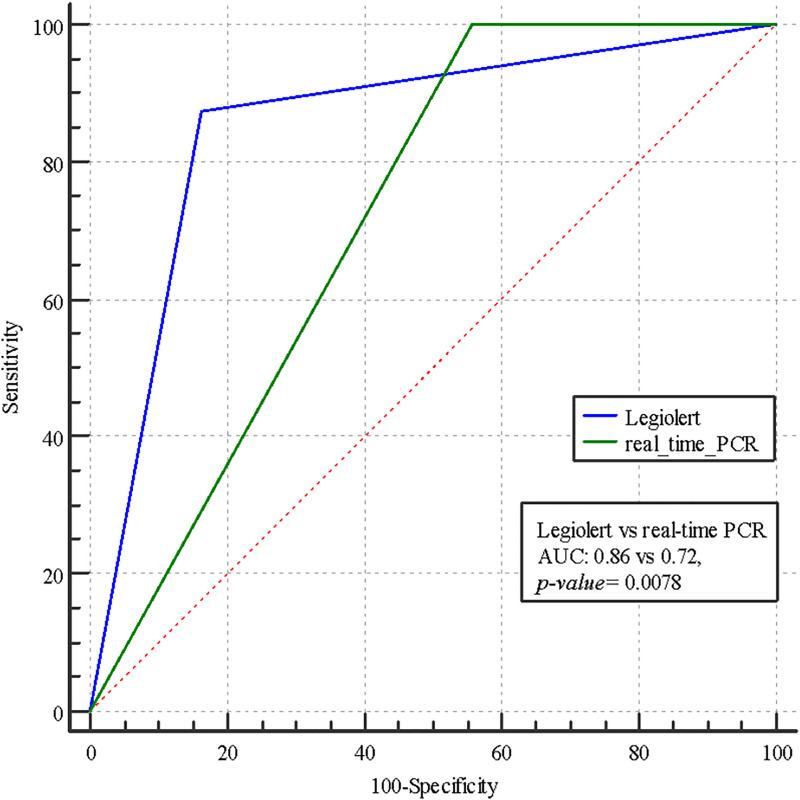
Comparison of ROC curves between real-time PCR and Legiolert.

Fig 4 shows that AUC associated with Legiolert was significantly greater than AUC associated with real-time PCR method (AUC: 0.86 vs 0.72, p = 0.0078), confirming that Legiolert was more accurate than real-time PCR method. Particularly, both methods had AUC values between 0.5 and 1, i.e., both methods were more accurate.

## 4. Discussion

Legionnaires, as already reported, is a pulmonary disease caused by inhalation of contaminated aerosol water droplets, and certainly the current methods used to detect and quantify *Lp* in water samples, which provide reliable results in the shortest possible time, are crucial for ensuring water quality and to prevent the microorganism diffusion. The method to detect the microorganism from water samples most widely used and considered as gold standard is the solid medium culture test but involves a lengthy incubation period of 10 days and needs for confirmatory testing in case of growth. Although the traditional plate culture method is regarded as the best practice, the above-mentioned limitations have prompted researchers to explore and develop alternative techniques. Indeed, the plate culture method remains time-consuming and requires considerable professional expertise, particularly for accurately identifying bacterial colonies [17]. The two solid media largely proposed GVPC and BCYE are selective equally and of high quality, studies aimed at evaluating bacterial detection have revealed that these media, especially GVPC, have low detection rates [18]. In contrast to these methods, the Legiolert test is straightforward to perform and provides final results within 7 days, offering high sensitivity and specificity, requiring further confirmation for the identification of *Lp* in water samples [19]. However, it is important to note that the Legiolert test exclusively detects *Lp* species. On this consideration, although infections caused by other *Legionella* species are very rare, it is essential to consider its potential for false-negative results [20]. The rapid turnaround time and high sensitivity of PCR offer clear advantages over culture methods, while specificity and accuracy were lower although the PCR positive results were higher than those obtained by plate culture and Legiolert. This discrepancy could be due to the ability that culture methods only quantify viable, cultivable and free *Lp,* potentially underestimating the *Lp* present within protozoa. In contrast, real-time PCR detects both viable and non-cultivable (VBNC) *Lp* and does not underestimate the *Lp* associated with protozoa [21]. However, nucleic acid in environmental samples can be highly stable and may persist for extended periods. Studies have shown that DNA from non-viable cells in biofilms can remain detectable from several days to weeks, depending on the microbial consortium present [22]. This study found that a high percentage of the buildings examined were colonized by *Lp.* According to the data presented here, Legiolert was able to detect more positive samples from 100 ml water rather than the ISO 11731 membrane filtration method which uses 1000 ml water, with sensibility, specificity and accuracy more than 80%, as described in the results. One possible explanation for the higher recovery rate of Legiolert compared to the standard method is that microorganisms may be more comfortable propagating in a liquid medium than in a solid one. This study further confirms the reliability of the Legiolert method in accordance with the gold standard and supports the use of real-time PCR as a detection tool to both exclude negatives and quickly identify positives. These findings align with previous studies that have demonstrated the consistency of Legiolert for detecting *Lp* in drinking water samples [23]. Our results suggest that *Lp* contamination was likely influenced by water temperature. Specifically, with the plate culture method, all *Lp*-positive samples came from water with temperatures higher than 55°C, aligning with other observations [24]. The classical culture method showed that approximately 51% of positive samples exceeded 10³ CFU/L, the threshold at which preventive measures should be considered. Instead, around 16% of the *Lp*-positive samples exceeded 10⁴ CFU/L, above the limit value for initiating decontamination measures according to Italian guidelines [16]. Several studies have indicated that *Lp* contamination levels above 10⁴ CFU/L at crucial sites can be considered high-risk for the transmission of Legionnaires’ disease [25]. This study demonstrated that the traditional culture method and Legiolert yielded similar results, confirming some advantages for Legiolert, including ease of reading and quantification, less technical expertise, and offering a shorter total evaluation time compared to the ISO method. Finally, it allows the result to be achieved with fewer resources. However, the use of molecular method considered in this study may have limitations, such as low *Lp* concentrations (e.g., > 35 cycles) representative of recently dead cells, viable but non-cultivable (VBNC) forms, or amoeba-encysted forms. In this case samples with low *Lp* concentrations are more challenging to interpret [26].

## 5. Conclusions

In conclusion, the findings from this study emphasize the need for certain hospitals, hotels and other accommodations in Sicily to implement preventive measures, as they pose a potential risk for Legionnaires’ disease. The study demonstrated that the culture method Legiolert offers several advantages over the conventional culture method, including ease of use, shorter time requirements, and fewer resources needed. While the plate culture method will remain an important detection tool, real-time PCR technique showed 100% of sensitivity according to the standard culture method results, enabling it to identify even low levels of contamination. This makes it a promising complementary tool to the standard culture-based approach for detecting *Lp* in water samples. Real-time PCR could be particularly valuable during a Legionnaires’ disease outbreak due to its high sensitivity, rapid acquisition of results and the easier handling of large sample amounts are further useful advantages. It is a profitable and beneficial supplementary tool to culture, particularly for screening negative samples. Our belief is that a molecular method that detects all *Legionella* spp could substitute traditional method in the assessment of the risk of infection. In conclusion, a limitation is that the high levels of *Lp* detected by PCR could represent viable but non-cultivable (VBNC) cells or DNA from lysed cells, so positive results should be carefully interpreted and may not always indicate an immediate health risk to vulnerable individuals.

## 6. Study limitations

In this study, the low number of positive samples reduced the ability to perform statistical analysis for some comparisons. Lastly, it is important to remember that data obtained from the real time PCR method only detects the presence of genetic material of the *Lp* species, as well as the legiolert test.
